# Is bruxism associated with the use of screen devices in children and adolescents? A systematic review

**DOI:** 10.1590/1807-3107bor-2026.vol40.054

**Published:** 2026-08-07

**Authors:** Ayah Qassem SHQAIR, Matheus dos Santos FERNANDEZ, Cacilda Castelo Branco LIMA, Marília Leão GOETTEMS, Francisco Wilker Mustafa Gomes MUNIZ

**Affiliations:** (a) Arab American University, Faculty of Graduate Studies, Department of Dental Sciences, Ramallah, Palestine.; (b) Universidade Federal de Pelotas – UFPel, Department of Periodontology, Pelotas, RS, Brazil.; (c) Universidade Federal do Piauí – UFPI, Postgraduate Program in Dentistry, Federal Teresina, PI, Brazil; (d) Universidade Federal de Pelotas – UFPel, Department of Social and Preventive Dentistry, Pelotas, RS, Brazil.

**Keywords:** Sleep Bruxism, Screen Time, Systematic Review, Adolescent, Child

## Abstract

This systematic review summarized findings on the association between screen time and bruxism in children and adolescents (PROSPERO- CRD42023429775). Two researchers conducted the search and selection of studies, extracted the data, and analyzed the risk of bias. The quality of the evidence was assessed using the GRADE tool. Five databases were screened on September 25^th^, 2025. Studies exploring the association between bruxism and the use of screen devices in individuals aged 0–18 were included, considering different and valid study designs, screen devices, and methods of detection of bruxism behavior with or without circadian distinction. Qualitative data synthesis was performed. Eleven cross-sectional and two cohort studies were included. Eight studies found an association between bruxism and the use of screen devices in children and adolescents, while five studies did not find a significant association. In conclusion, an association between screen use or duration and bruxism in children and adolescents cannot be established. Although some studies reported positive associations for awake, sleep, and unspecified bruxism, particularly in research evaluating longer daily screen time or indicators of problematic digital use, the certainty of the evidence was very low. Further well-designed longitudinal studies should be conducted to broaden the overview of this association.

## Introduction

Bruxism is a repetitive jaw-muscle activity characterized by clenching or grinding of the teeth and/or bracing or thrusting of the mandible. It has two distinct circadian manifestations: sleep bruxism (SB), which occurs during sleep, and awake bruxism (AB), which occurs during wakefulness.^1^ Bruxism is not classified as a movement or sleep disorder, but is rather seen as a motor behavior that may or may not be linked to clinical consequences such as dental wear.^1^ Bruxism activity is thought to originate from the central autonomic nervous system. The global prevalence of bruxism (sleep and awake) is 22.22%.^2^ The global prevalence of SB is 21% and AB is 23%. SB in childhood and adolescence ranges between 7.0% and 15.1%, with girls apparently more frequently affected.^3,4^

Bruxism can have a significant impact on the lives of children and adolescents, affecting their oral health and general well-being.^5,6^ Excessive pressure on the teeth can lead to tooth wear, dental sensitivity, jaw pain, and frequent headaches, impairing oral health-related quality of life.^7-9^ Sleep disturbances associated with nighttime bruxism can also result in daytime tiredness and difficulty concentrating.^10,11^ These consequences may be more severe in children due to the structural and morphofunctional characteristics of deciduous dentition.^12^ The etiology of bruxism is complex and multifactorial, with its pathophysiology still not fully understood.^13,14^ While AB is mainly attributed to psychosocial factors, the pathophysiology of SB appears more complex and centrally mediated.^15,16^ A meta-analysis examined factors linked to bruxism, emphasizing the significant role of emotional symptoms (e.g., nervousness and anger) and psychological disturbances (e.g., anxiety) in its occurrence among children.^7^ Other identified predictors include sex, genetic traits, sleep disorders, and habits like object biting (parafunctional) and sedentary behavior (*e.g.,* lack of physical activity and screen time).^7,17,18^

Screen time refers to the time spent on various types of screens, like watching television or movies, playing video games, or using smartphones and tablets.^19^ Cross-sectional studies have reported that over 40% of children engage in excessive screen time.^20,21^ Among adolescents, global estimates indicate that the prevalence of excessive screen time and television viewing was 70.9% and 58.8%, respectively.^22^ The association between screen time and bruxism in children and adolescents can be explained by several factors. Screen exposure may impact neurotransmitters, such as dopamine, and contribute to sleep disturbances and stress.^23^ Excessive screen time is linked to issues like longer sleep onset, insomnia, and poor sleep quality, which disrupt circadian rhythms and affect mood, cognitive development, and behavior.^24-26^ The main mechanisms include time displacement, psychological stimulation from media content, and the effects of light from screens on circadian rhythms and alertness.^27^ However, divergent results have been reported regarding the association between screen exposure and bruxism.^17,28,29^ Thus, a global synthesis of the available literature on the subject could provide a broader perspective on the impact of exposure to screen devices on the occurrence of bruxism. Therefore, this study aimed to systematically review the literature regarding the association between bruxism and the use of screen devices among children and adolescents.

## Methods

### Study design

This systematic review was reported following the Preferred Reporting Items for Systematic Reviews and Meta-analyses (PRISMA) 2020.^30^ The protocol of this systematic review was hosted on PROSPERO (CRD42023429775).^31^

### Review question and search strategy

This study was designed to gather evidence to answer the following question: ‘*Are the duration and/or use of screen devices associated with bruxism in children and adolescents?’.*

Search keywords were organized to identify studies of interest in five databases: PubMed, Scopus, Embase, Cochrane Library, and Web of Science. Full search strategies for these databases are available in Table 1. The search was last updated on September 25^th^, 2025. No limits were applied to publication date or original language of publication.

**Table 1:** Search strategy by databases..

.	
PubMed	#1 – Bruxism[Mesh Terms] OR Bruxism[Text Word] OR “Sleep bruxism”[Mesh Terms] OR “Teeth Grinding Disorder”[Text Word] OR Bruxomania[Text Word] OR “clenching teeth”[Text Word] OR “tooth clenching”[Text Word] OR “teeth clenching”[Text Word] OR “dental clenching”[Text Word]
(n = 437)	#2 – “Screen Time”[Mesh Terms] OR Screen [Text Word] OR Television [Mesh Terms] OR Television[Text Word] OR Televisions[Text Word] OR Smartphone [Mesh Terms] OR Smartphone[Text Word] OR Smartphones[Text Word] OR “Smart Phone”[Text Word] OR “Smart Phones”[Text Word] OR “Mobile Applications”[Mesh Terms] OR “Mobile applications”[Text Word] OR Mobile[Text Word] OR App[Text Word] OR Application[Text Word] OR Applications[Text Word] OR “Cell Phone Use”[Mesh Terms] OR “Cell phone”[Text Word] OR “Video Games”[Mesh Terms] OR “Video game”[Text Word] OR “Video games”[Text Word] OR “Computer Game”[Text Word] OR “Computer games”[Text Word] OR “Computers”[Mesh Terms] OR Computer[Text Word] OR Computers[Text Word] OR Microcomputer[Text Word] OR Microcomputers[Text Word] OR Tablet[Text Word] OR Tablets[Text Word] OR Media[Text Word] OR Laptop [Text Word] OR Gadgets [Text Word] OR Ipad [Text Word] OR Iphone [Text Word]
	#3 – #1 AND #2
Web of Science	#1 – TS=(Bruxism OR “Sleep bruxism” OR “Teeth Grinding Disorder” OR Bruxomania OR “clenching teeth” OR “tooth clenching” OR “teeth clenching” OR “dental clenching”)
(n = 585)	#2 – TS=(“Screen Time” OR Screen OR Television OR Televisions OR Smartphone OR Smartphones OR “Smart Phone” OR “Smart Phones” OR “Mobile applications” OR Mobile OR App OR Application OR Applications OR “Cell phone” OR “Video game” OR “Video games” OR “Computer game” OR “Computer games” OR Computer OR Computers OR Microcomputer OR Microcomputers OR Tablet OR Tablets OR Media OR Laptop OR Gadgets OR Ipad OR Iphone)
	#3 – #1 AND #2
Scopus	#1 – TITLE-ABS-KEY (Bruxism OR “Sleep bruxism” OR “Teeth Grinding Disorder” OR Bruxomania OR “clenching teeth” OR “tooth clenching” OR “teeth clenching” OR “dental clenching”)
(n = 896)	#2 – TITLE-ABS-KEY (“Screen Time” OR Screen OR Television OR Televisions OR Smartphone OR Smartphones OR “Smart Phone” OR “Smart Phones” OR “Mobile applications” OR Mobile OR App OR Application OR Applications OR “Cell phone” OR “Video game” OR “Video games” OR “Computer game” OR “Computer games” OR Computer OR Computers OR Microcomputer OR Microcomputers OR Tablet OR Tablets OR Media OR Laptop OR Gadgets OR Ipad OR Iphone)
	#3 – #1 AND #2
Embase	#1 – (Bruxism:ti,ab,kw OR “Sleep bruxism”:ti,ab,kw OR “Teeth Grinding Disorder”:ti,ab,kw OR Bruxomania:ti,ab,kw OR “clenching teeth”:ti,ab,kw OR “tooth clenching”:ti,ab,kw OR “teeth clenching”:ti,ab,kw OR “dental clenching”:ti,ab,kw)
(n = 409)	#2 – (“Screen Time”:ti,ab,kw OR Screen:ti,ab,kw OR Television:ti,ab,kw OR Televisions:ti,ab,kw OR Smartphone:ti,ab,kw OR Smartphones:ti,ab,kw OR “Smart Phone”:ti,ab,kw OR “Smart Phones”:ti,ab,kw OR “Mobile applications”:ti,ab,kw OR Mobile:ti,ab,kw OR App:ti,ab,kw OR Application:ti,ab,kw OR Applications:ti,ab,kw OR “Cell phone”:ti,ab,kw OR “Video game”:ti,ab,kw OR “Video games”:ti,ab,kw OR “Computer game”:ti,ab,kw OR “Computer games”:ti,ab,kw OR Computer:ti,ab,kw OR Computers:ti,ab,kw OR Microcomputer:ti,ab,kw OR Microcomputers:ti,ab,kw OR Tablet:ti,ab,kw OR Tablets:ti,ab,kw OR Media:ti,ab,kw OR Laptop:ti,ab,kw OR Gadgets:ti,ab,kw OR Ipad:ti,ab,kw OR Iphone:ti,ab,kw)
	#3 – #1 AND #2
Cochrane	#1 – Bruxism OR “Sleep bruxism” OR “Teeth Grinding Disorder” OR Bruxomania OR “clenching teeth” OR “tooth clenching” OR “teeth clenching” OR “dental clenching”
(n = 167)	#2 – “Screen Time” OR Screen OR Television OR Televisions OR Smartphone OR Smartphones OR “Smart Phone” OR “Smart Phones” OR “Mobile applications” OR Mobile OR App OR Application OR Applications OR “Cell phone” OR “Video game” OR “Video games” OR “Computer game” OR “Computer games” OR Computer OR Computers OR Microcomputer OR Microcomputers OR Tablet OR Tablets OR Media OR Laptop OR Gadgets OR Iphone OR Ipad
	#3 – #1 AND #2
Google Scholar	Bruxism AND Screen
(n = 300)	

The “PECO(S)” strategy was used, which included the following criteria:

(P)opulation: individuals with aged ≤18 years old;

(E)xposure: individuals exposed to any screen devices - *e.g.,* television, tablet, mobile phone, computer and others; or duration of exposure;

(C)omparison: individuals who were not exposed to any screen devices or exposed for a short period of time;

(O)utcome: occurrence of sleep or awake bruxism;

(S)tudy design: cross-sectional, case-control, or cohort studies.

Results of literature searches were uploaded to EndNote Web (https://web.endnote.com/; Thomson Reuters, New York, USA), and duplicate records were automatically removed by one researcher (AQS). Two researchers (AQS and MLG) screened all titles and abstracts, considering the selection criteria using Rayyan – a website for systematic reviews (https://www.rayyan.ai/). The kappa (κ) agreement between researchers was calculated. Discrepancies were resolved by discussion with a third reviewer (FWMGM).

A manual search was conducted of issues published in the last 10 years in the two main journals on children’s and adolescents’ oral health: *International Journal of Paediatric Dentistry* (https://onlinelibrary.wiley.com/journal/1365263X) and *European Journal of Pediatric Dentistry* (https://www.ejpd.eu/). The search was also performed in the Google Scholar database using an adapted search strategy (Bruxism AND Screen); only the first 300 studies were screened for eligibility. All relevant studies, including grey literature (theses and dissertations) and peer-reviewed articles, were also eligible for inclusion from the *Google Scholar* database. The list of references of all the studies selected after full-text reading was screened to identify records not included in the previous searches.

### Eligibility criteria

The following criteria were considered for the inclusion of studies in this systematic review:

- Studies with children and adolescents aged 0–18 years;
- Studies investigating the association between bruxism and screen use, defined as activities performed in front of one or multiple types of screen-based devices (e.g., television, smartphone, tablet, laptop, desktop computer, video games, and similar devices);
- Cross-sectional, cohort, and case-control studies;
- Bruxism assessed by parental/self-report questionnaires, clinical examination, and/or instrumental assessment tools based on electromyography or polysomnography.

Studies limited to participants with special health care needs, defined as individuals with physical, developmental, mental, sensory, behavioral, cognitive, or emotional impairments, were excluded.

### Data extraction and synthesis of the evidence

A data extraction spreadsheet, based on the Cochrane Consumers and Communication Review Group’s data extraction template, was adapted for this study using Microsoft Excel. Two reviewers (AQS and MSF) extracted all data from the included studies, and both researchers checked the data jointly; potential discrepancies were assessed and verified as needed. The following information was collected: author, main author email, year, type of study, country, objective, aspects of the sample [(n), age, sex], bruxism assessment, screen device use measurement, main results [univariate, bivariate, and multivariate analysis], conclusion/highlights, use of guidelines [https://www.equator-network.org/reporting-guidelines/strobe/, yes *vs.* no], open science practices [open access, protocol index and data sharing, yes *vs.* no]. In case of any missing data, the corresponding authors of the included studies were contacted by e-mail to provide additional information for to be included in the quantitative analysis. A total of eight authors were contacted. Authors who did not reply to requests were contacted twice more after three and six weeks via email and their ResearchGate profile - https://www.researchgate.net/ (response rate: 0%).

### Risk of bias

The Joanna Briggs Institute’s (JBI) Critical Appraisal Checklist for cross-sectional studies (8 items) was used to identify the risk of bias of the included records. The answers to this tool were “yes”, “no”, “unclear,” or “not applicable”.

The risk of bias of the included cohort studies was assessed with the Newcastle-Ottawa Scale (N-OS). The N-OS contains eight items categorized into three dimensions: selection, comparability, and results. A scoring system was used for the semi-quantitative assessment of study quality. Each study was awarded one star for each item, except for comparability, which could receive up to two stars. The maximum possible score was nine.

Two researchers (AQS and CCBL) analyzed the risk of bias, and disagreements between the reviewers in this step were resolved by discussion with a third reviewer (FWMGM). The κ agreement between the examiners was determined. Due to the low number of included studies, the impact of high risk of bias for the overall estimates was not feasible in this study.

### Certainty of evidence

The Grading of Recommendations Assessment Development and Evaluation (GRADE) tool (www.gradepro.org/) was used to assess the certainty of evidence.^32^ This assessment was performed by one researcher (FWMGM). The risk of bias, inconsistency, indirectness, imprecision, and other methodological and sampling aspects of the studies were considered to determine the certainty of the evidence. The summary of findings was prepared in a narrative data synthesis.

## Results

### Study selection process

Searches in all electronic databases yielded 2,494 potentially relevant records. The manual search of journals did not identify any additional records for inclusion. A total of 1,177 duplicates were removed. Subsequently, 1,317 records were screened by title and abstract. Of these, 15 full-text articles were read, with two excluded [age group (n = 1); did not clearly show the results of the association between screen use and bruxism (n = 1)] and thirteen included^17,28,33-43^ (Figure 1*)*. Kappa coefficients between the two researchers were κ = 0.88 and κ = 1.00 for title/abstract and full-text selection steps, respectively, indicating a substantial agreement.

**Figure:**
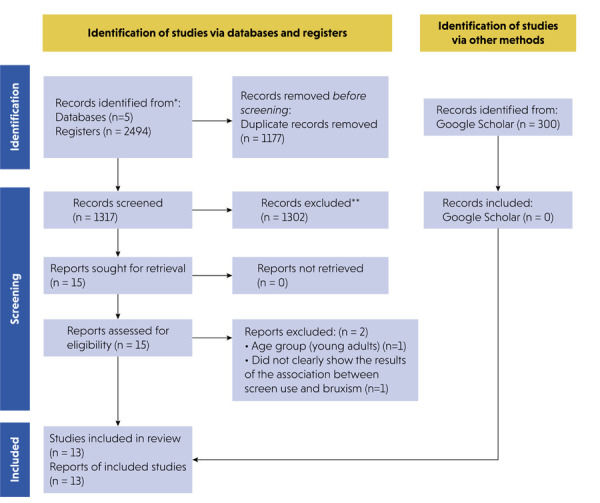
PRISMA 2020 flow diagram for systematic reviews, which included searches of databases, registers and other sources.

### Risk of bias of included studies

The risk of bias assessment for the eleven included cross-sectional studies indicated that most of the registries (n = 8) received “yes” to all questions, indicating a low risk of bias^17,33,36-41^ (Table 2). Regarding the cohort studies, a risk of bias was detected in the “demonstration that outcome of interest was not present at start of study” in both studies^28,34^ (Table 3). Meanwhile, additional risk of bias (comparability and adequacy of follow-up of cohorts) was detected in one study.^28^ The Kappa coefficient between the researchers was κ = 0.97 and κ = 0.92 for cross-sectional studies and cohort study, respectively, indicating substantial level of agreement.

**Table 2:** Assessment of the risk of bias in cross-sectional studies.

Author (Year)	JBI for analytical cross-sectional studies								Additional comments, if applicable
	Q1	Q2	Q3	Q4	Q5	Q6	Q7	Q8	
Alanzi et al. (2025)^37^	Yes	Yes	Yes	Yes	Yes	Yes	Yes	Yes	
Al-Swaje et al. (2019)^46^	No	No	No	Yes	NA	NA	Yes	No	Q1: It does not present inclusion and exclusion criteria. Q2: The study participants are not described in detail, and the time period was not clear. Q3: The exposure was not measured in a valid and reliable way. Q8: The data were analyzed using only the Chi-square test.
Amaral et al. (2022)^21^	Yes	Yes	Yes	Yes	Yes	Yes	Yes	Yes	
Meenapriya et al. (2021)^47^	No	No	Yes	Yes	NA	NA	Yes	No	Q1: It does not present inclusion and exclusion criteria. Q2: The study participants are not described in detail. Q8: The data were analyzed using only the Chi-square test.
Güzel et al. (2025)^39^	Yes	Yes	Yes	Yes	Yes	NA	Yes	No	Q8: No regression analyses were performed.
Marceliano; Gavião (2023)^40^	Yes	Yes	Yes	Yes	Yes	Yes	Yes	Yes	
Pauli et al. (2024)^41^	Yes	Yes	Yes	Yes	Yes	Yes	Yes	Yes	
Prado et al. (2025)^42^	Yes	Yes	Yes	Yes	Yes	Yes	Yes	Yes	
Restrepo; Santamaría; Manrique (2021)^43^	Yes	Yes	Yes	Yes	NA	NA	Yes	Yes	
Silva et al. (2022)^44^	Yes	Yes	Yes	Yes	Yes	Yes	Yes	Yes	
Yazıcıog˘ lu et al. (2021)^45^	Yes	Yes	Yes	Yes	Yes	Yes	Yes	Yes	

Q1: Were the criteria for inclusion in the sample clearly defined?; Q2: Were the study subjects and the setting described in detail?; Q3: Was the exposure measured in a valid and reliable way?; Q4: Were objective, standard criteria used for measurement of the condition?; Q5: Were confounding factors identified?; Q6: Were strategies to deal with confounding factors stated?; Q7: Were the outcomes measured in a valid and reliable way?; Q8:Was appropriate statistical analysis used?. NA: Not applicable.

**Table 3:** Analysis of the risk of bias of the included cohort studies.

Newcastle - Ottawa quality assessment scale cohort studies	Carrillo-Diaz et al., (2021)^32^	Chen, Gau, (2016)^38^
Selection		
1) Representativeness of the exposed cohort	★	
2) Selection of the non-exposed cohort	★	
3) Ascertainment of exposure	★	
4) Demonstration that outcome of interest was not present at start of study		
Comparability		
1) Comparability of cohorts on the basis of the design or analysis		
Outcome	★	
1) Assessment of outcome	★	
2) Was follow-up long enough for outcome to occur		
3) Adequacy of follow-up of cohorts		

### Qualitative analyses of the evidence

#### Study characteristics

The methodological descriptions of the studies included in this review are presented in Table 4. Eleven of the included studies had a cross-sectional design, while two were conducted as cohort studies.^28,34^ The studies were conducted in different countries: Brazil (n = 5),^17,36-38,40^Colombia (n = 1),^39^ India (n = 1),^43^ Kuwait,^32^ Spain (n = 1),^28,34^ Taiwan (n = 1),^34^ Turkey (n = 2),^35,41^ and Saudi Arabia (n = 1).^42^ In total, 8,315 individuals from the thirteen studies were included.

**Table 4:** Methodological aspects of the included studies.

Author, Year; Country	Study design	Sample (n); age; sex	Bruxism assessment	Screen device use assessment
Alanzi et al., 2025^37^; Kuwait	Cross-sectional	388 children aged 3-5 years; 188 boys and 200 girls	Maternal report: yes vs no	Problematic Media Use Measure-
Al-Swaje et al., 2019^46^; Saudi Arabia	Cross-sectional	204 children aged 5-10 years; 50 boys and 154 girls	Tooth wear: positive vs negative	Smart device or play station possession (yes vs. no), duration of smart device possession (≤2 vs >2 years), weekly frequency of usage (1-2 vs 3-4 vs >4 days/week), and daily frequency of usage (≤2 vs 3-5 vs >5hours).
Amaral et al., 2022^21^; Brazil	Cross-sectional	556 children aged 7-8 years; 288 boys and 268 girls	Probable sleep bruxism (positive clinical inspection with/without a positive parental report): yes vs no	Total screen time (≤2 h/day vs. >2 to 3 h/day vs. >3 to 4 h/day vs. >4 to 5 h/day vs. >5 h/day).
Carrillo-Diaz et al., 2021^32^; Spain	Cohort	213 adolescents aged 11-17 years; 116 girls and 97 boys	Self-reported Bruxism Questionnaire (SBQ) (ranging 11-55 points)	CERI (Questionnaire of Experiences Related to Internet Use and (Questionnaire of Experiences Related to Cell Phones (CERM): a range of 10-40 points, higher scores indicate higher levels of internet and telephone use.
Chen; Gau, 2016^38^; Taiwan	Cohort	1253 children and adolescents; The age ranges were 8–12 years (grade 3 students), 10–12 years (grade 5 students), 13–16 years (grade 8 students); 615 girls and 638 boys.	Self-report of bruxism: yes vs no	Chen Internet Addiction Scale (CIAS) assesses internet addiction severity, with higher scores indicating greater addiction.
Meenapriya et al., 2021^47^; India	Cross-sectional	300 children aged 4-6 years; 160 boys and 140 girls.	Parental report (yes vs no) and clinical examination (abnormal wear and discomfort of the jaws)	Time of gadget usage (>2hour vs 1-2 hours vs <1hour).
Güzel et al., 2025^39^; Turkey	Cross-sectional	300 adolescents aged 10–19 years; 151 males and 149 females	Self-reported questionnaires (American Academy of Sleep Medicine) for sleep bruxism and awake bruxism: yes vs no	Video game addiction (yes vs no), according to the Digital Game Addiction Scale −21 (DGAS-21)
Marceliano; Gavião, 2023^40^; Brazil	Cross-sectional	174 children aged 6-14 years; 99 girls and 79 boys	Possible sleep bruxism (parent report): without, sometimes and frequent	Screen time–working days (hours) and Screen time–weekend (hours)
Pauli et al, 2024^41^; Brazil	Cross-sectional	3,229 children aged 4 years; 1646 girls and 1589 boys	Possible sleep bruxism (parental report): yes vs no	Screen time (hours per day): Excessive (≥ 2h) vs Normal (<2)
Prado et al, 2025^42^; Brazil	Cross-sectional	403 adolescents aged 11–19 years; 234 girls and 169 boys	Possible sleep bruxism (self-report grinding, bracing and thrusting activity): absent vs mild vs moderate severe activity	Smartphone use (daily hours, use in bed before bedtime, upon waking, in bed with lights off, etc.
Restrepo, Santamaría, Manrique, 2021^43^; Colombia	Cross-sectional	460 children aged 4-8 years; NR	Possible sleep bruxism (parental report): never rarely vs sometimes vs usually	Mean hours per day spent using screens (cell phones,
Silva et al., 2022^44^; Brazil	Cross-sectional	739 children aged 8-10 years; 367 boys and 372 girls	Possible awake bruxism (self-report): yes vs no	Daily use of electronic devices (yes vs no);
Yazıcıoglu et al., 2021^45^; Turkey	Cross-sectional	96 children; 8.3 (SD: 1.4) years; 52 girls and 44 boys	Parent-reported (American Academy of Sleep Medicine): yes vs no	Television time (last 3 months) – hours;

Univariate and bivariate analyses were the statistical methods used in three studies.^41-43^ Adjusted analysis models were used in the other included studies (n = 10) to verify the association between bruxism and exposure to screen devices.

#### Bruxism definition and assessment approach

The assessment of bruxism occurrence varied across the studies (Table 4). Most of the included studies evaluated bruxism using parent-reported questionnaires^33,36,37,39,41^ or self-reported questionnaires.^34,35,38,40^ Two studies evaluated the presence of clinical signs, such as wear facets,^17,42^ and two studies used both assessment modes (subject-based and clinically based).^28,43^ None of the included studies employed device-based assessment tools such as polysomnography (PSG), audio-video recordings, or electromyography (EMG).

Regarding bruxism manifestations, nine studies evaluated sleep bruxism,^17,28,,34,36-39,41,43^ one evaluated awake bruxism,^40^ one evaluated both manifestations,^35^ and two did not specify the manifestation.^33,42^

#### Screen use measurement

Screen use was assessed using heterogeneous approaches across the included studies, with substantial variability in measurement tools, device types, reporting methods, and exposure categories. The majority of studies screen time, quantified as the duration of exposure in hours per day, mean hours across weekdays and weekends, or categorized thresholds such as < 1 h, 1–2 h, and ≥ 2 h per day. In these studies, excessive screen time was defined as screen use exceeding pediatric guideline recommendations or as a higher level of screen exposure compared with a control or reference group with lower screen time.

In four studies, screen use was evaluated using validated instruments designed to assess problematic or addictive digital behaviors.^28,33,34^ These instruments typically evaluated excessive media usage that disrupts normal functioning and generated scores, with higher scores or categories indicating greater levels of screen engagement or problematic use.

Some studies further differentiated exposure by device type, including televisions, smartphones, tablets, computers, and video games, while others aggregated screen use across multiple devices.

#### Synthesis of results

The included studies demonstrated heterogeneous results, but generally found significant associations between screen use and higher occurrence of bruxism in children and adolescents. Of the thirteen studies included in this review, eight found an association between bruxism and the use of screen devices in children and adolescents,^28,33,35,37,39,40,42,43^ while five studies did not report a significant association.^17,34,36,38,41^ Regarding bruxism manifestation, of the ten studies that evaluated sleep bruxism, five found an association with the use of screens among adolescents. ^28,35,37,39,43^Awake bruxism and unspecified bruxism were associated with screen use in all the studies.

Longer duration of use in hours was associated with a higher occurrence of bruxism in five of the nine studies that evaluated usage in hours.^37,39,40,42,43^ Three studies^28,33,34^ found a higher occurrence of bruxism and screen use when assessed by questionnaires that evaluate problematic digital use or internet addiction, while one study^34^ did not find a statistically significant association.

#### Certainty of evidence

Table 5 presents the GRADE assessment for the qualitative analysis conducted in this study, revealing a very low certainty of evidence. The main reasons for downgrading were study limitations, primarily due to a serious risk of bias (moderate to high risk), significant inconsistency (high heterogeneity across analyses), and imprecision (very small sample size in the included studies with wide 95% confidence intervals detected in several studies that provided this estimate).

**Table 5:** *GRADE Tool*: certainty analysis of the evidence from qualitative synthesis performed in this study.

Certainty assessment							Impact	Certainty	Importance
Nº of studies	Study design	Risk of bias	Inconsistency	Indirectness	Imprecision	Other considerations			
Bruxism (outcome)									
13	Non-randomized studies	Serious^a^	Serious^b^	Very serious^c^	Very serious^c,d^	None	Four studies used only univariate and bivariate analyses as statistical methods. In eight studies, robust adjusted models were applied to investigate the association between bruxism behavior and exposure to screen devices. Among the 13 studies included in this review, only eight identified an association between the use of screen devices and bruxism in children and adolescents.	⨁◯◯◯	Critical
								Very low^c,d,e^	

a. Of the total number of cross-sectional studies included (n = 11), eight received “yes” to all criteria assessed. Among the cohort studies (n = 2), the maximum number of stars (which indicated low risk of bias) was not attributed to both of them; b. Studies with several ways to diagnose bruxism (outcome). In addition, several ways to assess screen devices excessive use was observed among the included studies; c. The global estimated effect analysis of the association between bruxism behavior and exposure to screen devices was not performed. Different diagnostic and classification methods for the outcome and main exposure were used in the included studies, and divergent results were identified. Therefore, the evidence presented in this study is considered indirect; d. Studies with small samples. In addition, only nine studies presented data variability to be assessed (e.g. 95% confidence interval), of which five of them were considered as very wide.

## Discussion

This study was designed to answer the following question: *“Are the duration and/or use of screen devices associated with bruxism in children and adolescents?”* The global synthesis of the thirteen studies indicates conflicting results, with over half of the studies linking screen use and/or duration with bruxism in children and adolescents. Despite considerable variability in assessment criteria and screen-use measurement, all available studies on awake and unspecified bruxism showed positive results. Evidence for sleep bruxism was mixed, with associations observed in five of ten studies. Studies assessing longer daily screen time or higher levels of problematic use more often reported positive associations. However, given the substantial methodological limitations and the very low certainty of evidence, these findings should be interpreted cautiously.

Based on a literature review performed by the authors, this is the first systematic review to explore the proposed topic. Some psychological and physiological theories may explain the relationship between exposure to screen time and bruxism. Excessive screen time can affect neurotransmitters and contribute to higher stress levels, disturb sleep patterns,^44^ and disrupt biological rhythm.^27^ The relationship between bruxism, stress, sleep disorders, and biological rhythms among children and adolescents is complex and disruption of this balance may contribute to bruxism occurrence.^45^

Detection of bruxism can be complex, and the selection of assessment method depends on the specific research question, as self-reporting, clinical examination, and device-based tools each assess different aspects of bruxism.^1^ Most included studies relied on parent- or self-reported questionnaires. Subjective reports are prone to recall bias and limited awareness of behaviors, but they allow the assessment of the perceived time course of bruxism.^1^ A minority of studies relied on clinical examination or used a combination of both assessment methods. Notably, clinical examination does not directly measure bruxism; instead, it identifies clinical signs of motor behavior that may be occur independently of patients’ perceptions (e.g., tongue impressions) and may reflect past activity rather than current behavior (e.g., tooth wear).^1^ Consequently, clinical indicators like tooth wear are not specific to active bruxism and may result from past behaviors or other factors such as diet, developmental defects, or normal physiological processes. Therefore, the interpretation of our findings should consider the heterogeneity in bruxism assessment methods used across studies. Future research should adopt tools aligned with international consensus recommendations to improve comparability.

The American Academy of Pediatrics recommends that children over age five through adolescence have less than two hours of screen time per day. However, a large percentage of children and adolescents already exceed this recommendation,^46^ making it a sedentary habit. In general, these media-related activities occupy about 6 to 9 hours per day, excluding housework and schoolwork.^20,22^ The impact of sedentary screen device use on the general health of children and adolescents has been explored in the literature.^47,48^ Therefore, all pediatric professionals need to encourage families to reduce the amount of time children and adolescents spend using screen devices. Removing electronic media from bedrooms, including televisions, video games, computers, tablets, and mobile phones, can also be suggested, considering all possible consequences for systemic and oral health.

In general, studies assessed exposure to various screen devices based on the average number of hours of exposure during workdays or weekends, or on the prevalence of excessive exposure to the devices (dichotomous variable), defined according to criteria suggested by the WHO^49^ and the American Academy of Pediatrics.^50^ Most studies consider overall exposure to screen devices without differentiating between device types or the activities performed during screen time, which could provide new insights into patterns of exposure to screen devices and oral health outcomes, especially concerning bruxism. For example, playing video games on screen devices is common among children aged 8 to 17; estimates indicate this population group spends an average of 1.5 to 2 hours daily playing video games.^52^

Methodological limitations contributed substantially to the very low certainty of the evidence. Potential biases must also be considered. Selection bias may have influenced results in studies using convenience samples, and exposure misclassification is likely when studies fail to distinguish between different device types or purposes of screen use (e.g., educational versus recreational). Most studies assessed total screen time rather than specific patterns or contexts, which limits the understanding of whether certain activities (e.g., gaming, social media, or nighttime viewing) are more strongly related to bruxism. The lack of other searches in the grey literature must also be faced as another limitation, as this may reduce publication bias, as this may be a source of publication bias.

Different tools were used to assess the risk of bias in the present study. For cohort studies, the N-OS scale was used as proposed in the literature.^52^ However, the JBI scale can also be used for cohort studies. Both scales are very similar, but the JBI additionally assesses aspects, such as reasons for loss to follow-up, strategies used by studies to address incomplete follow-up, and if appropriate statistical analyses were performed. Readers should note that these three aspects were not assessed in the present study, which may be considered a limitation.

The certainty of evidence in this field is very low, mainly due to cross-sectional designs, small sample sizes, and inconsistent measurement approaches. Therefore, results should be interpreted with caution. To strengthen future research, longitudinal studies with representative samples are needed, using validated tools and objective measures of screen exposure (such as digital logs and monitoring apps). Differentiating device type and activity context may also clarify behavioral patterns associated with bruxism risk. Moreover, adopting open science practices—such as protocol registration, data sharing, and transparent reporting—will enhance reproducibility and meta-analytic potential.

## Conclusion

In conclusion, an association between screen use or duration and bruxism in children and adolescents cannot be established. Although some studies reported positive associations for awake, sleep, and unspecified bruxism, particularly in research evaluating longer daily screen time or indicators of problematic digital use, the certainty of the evidence was very low. Future studies should use standardized assessment methods, objective measures of screen exposure, and longitudinal designs to clarify this relationship.

## Data Availability

The datasets generated during and/or analyzed during the current study are available from the corresponding author on reasonable request.
